# Supplementary material for: Perceptual super-resolution in multiple sclerosis MRI
Source: Front Neurosci. 2024 Oct 22;18:1473132. doi: 10.3389/fnins.2024.1473132 (PMC11534588; doi:10.3389/fnins.2024.1473132)
Supplement: Supplementary file 1 [file Data_Sheet_1.pdf]

Supplementary Material

1 SUPPLEMENTARY FIGURES

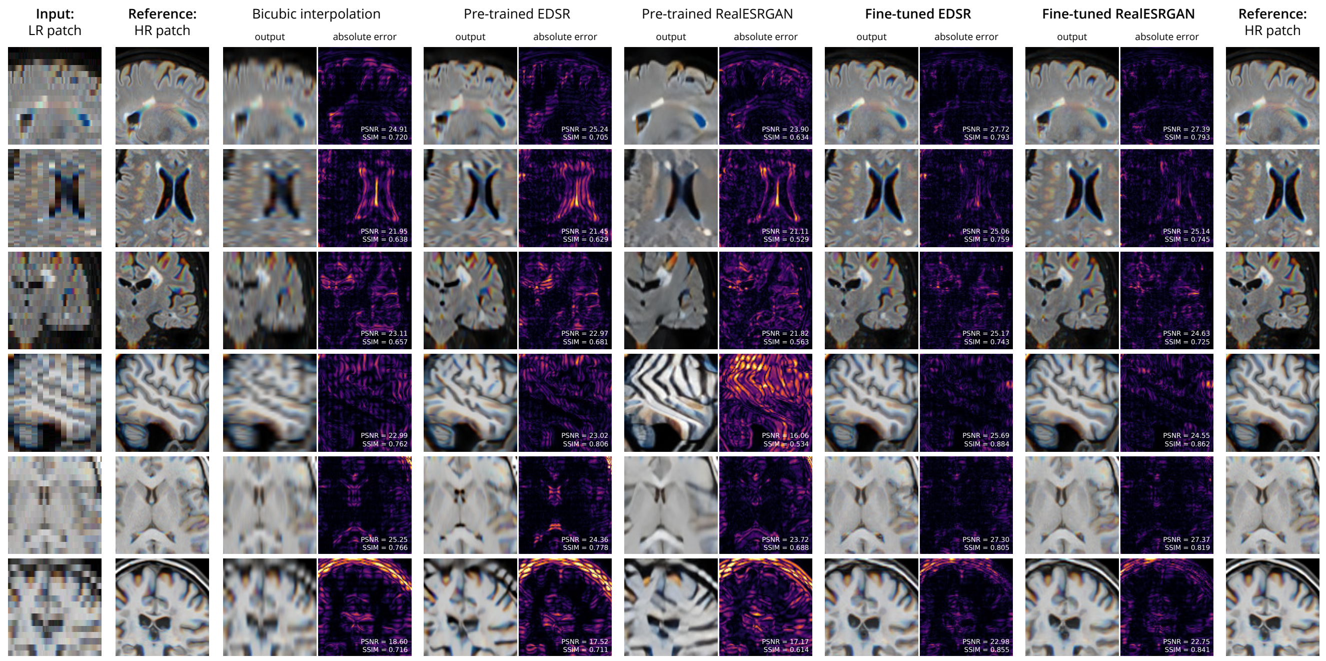

Figure S1. Examples of paired LR-HR patches, bicubic interpolation applied to LR patches, and patch super-resolution with pre-trained and fine-tuned CNN models. Patches are shown as RGB images where each color channel represents one of three contiguous patches in the third dimension.

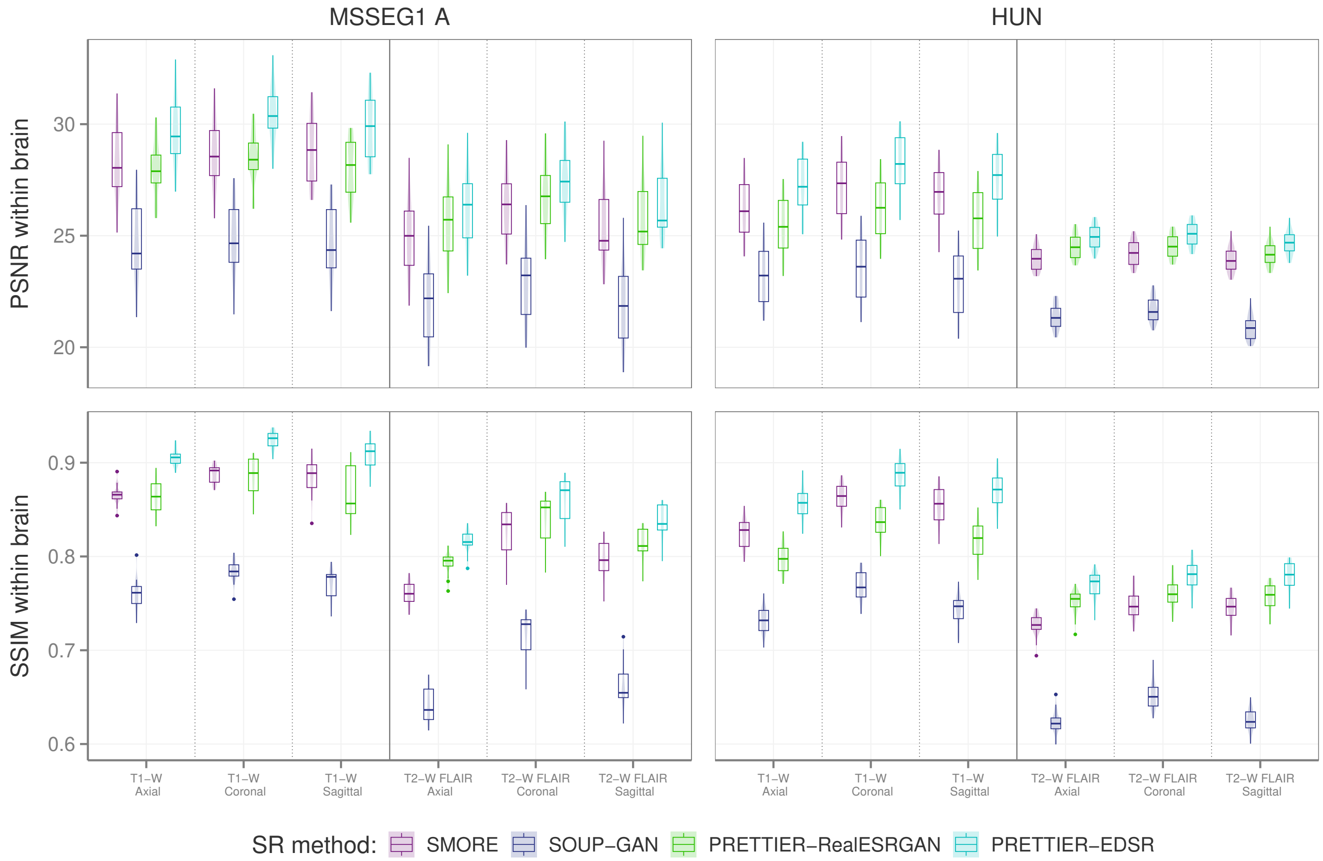

Figure S2. PSNR and SSIM distributions for reconstruction of MRI volumes in the evaluation set.

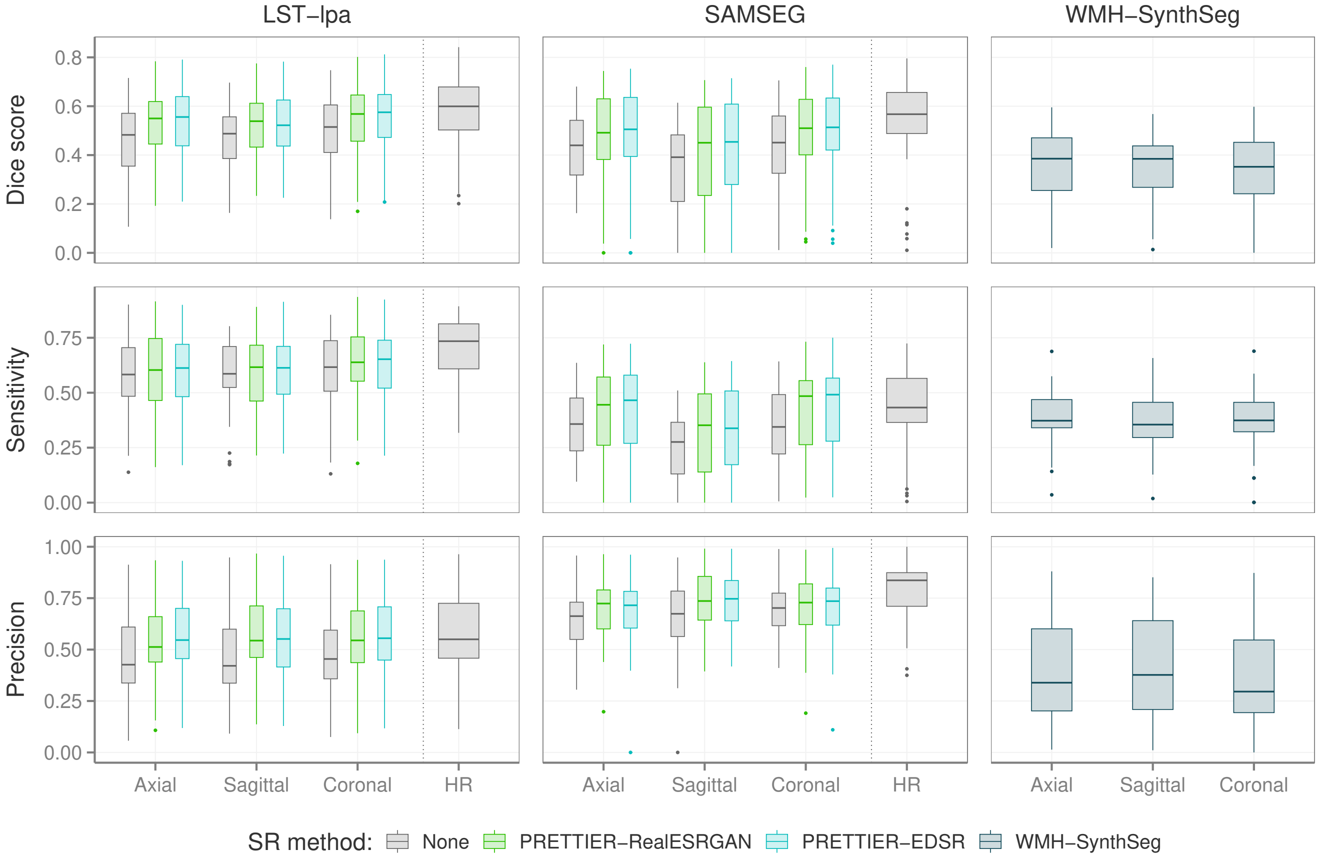

Figure S3. Distributions of Dice score, sensitivity, and precision for automated lesion segmentation on T2-W FLAIR images with LST-lpa, SAMSEG and WMH-SynthSeg.

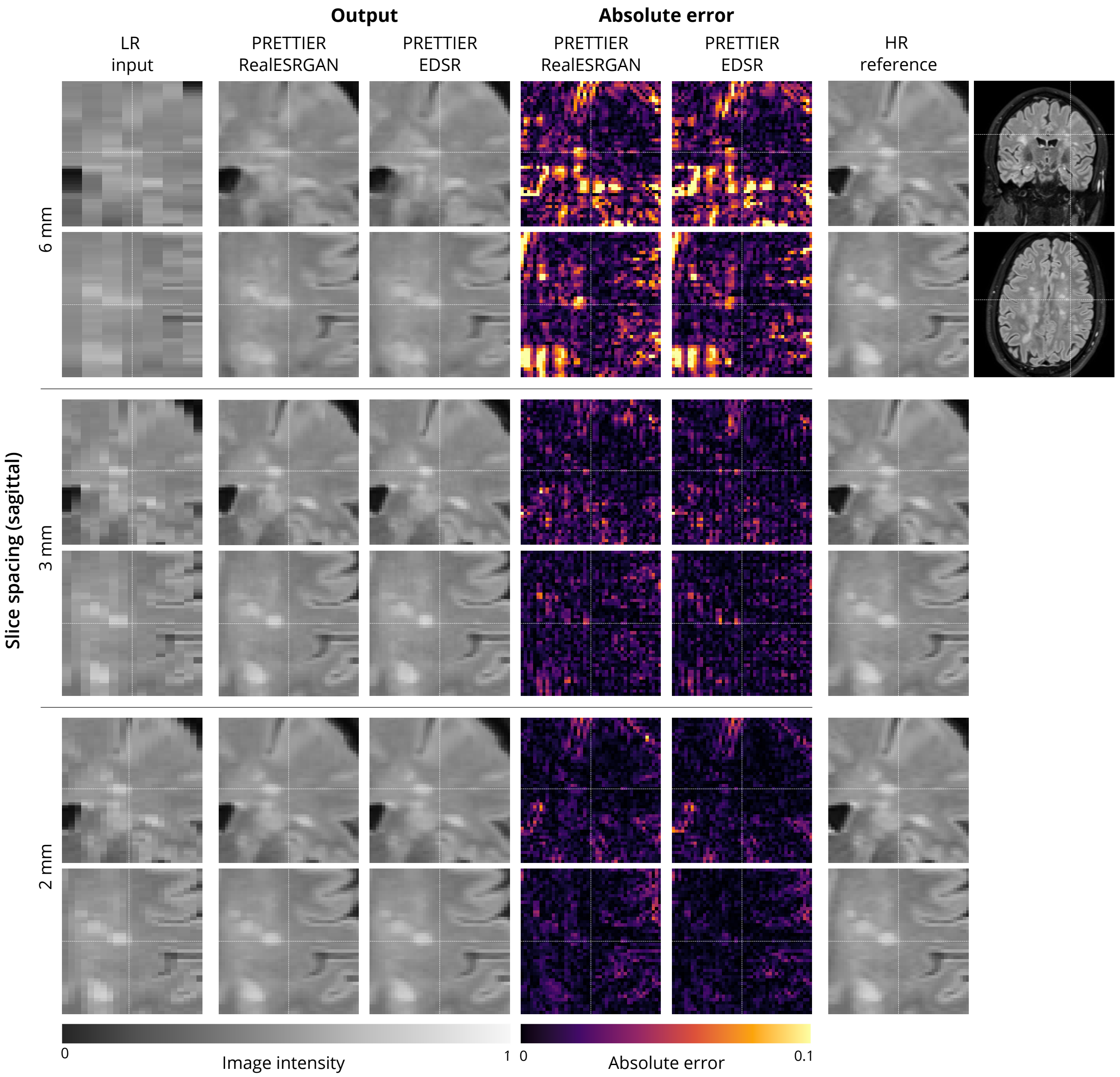

Figure S4. Qualitative results of PRETTIER SR reconstructions from LR T2-W FLAIR, simulated with sagittal slice orientation and three different slice profiles: 6 mm of slice spacing with 1 mm of slice gap (top panel), 3 mm of slice spacing without slice gap (middle panel), and 2 mm of slice spacing without slice gap (bottom panel).

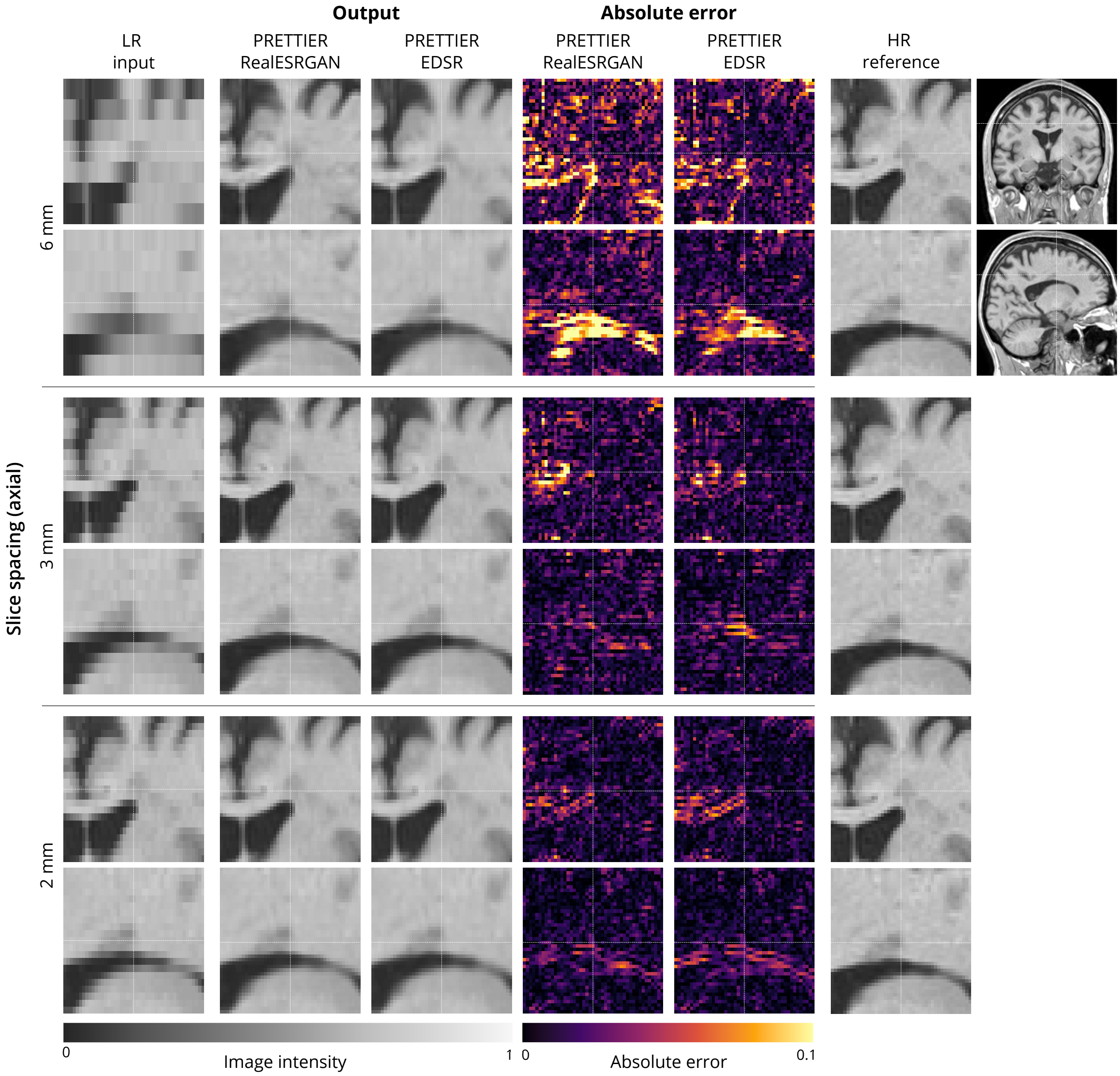

Figure S5. Qualitative results of PRETTIER SR reconstructions from LR T1-W MRI, simulated with axial slice orientation and three different slice profiles: 6 mm of slice spacing with 1 mm of slice gap (top panel), 3 mm of slice spacing without slice gap (middle panel), and 2 mm of slice spacing without slice gap (bottom panel).

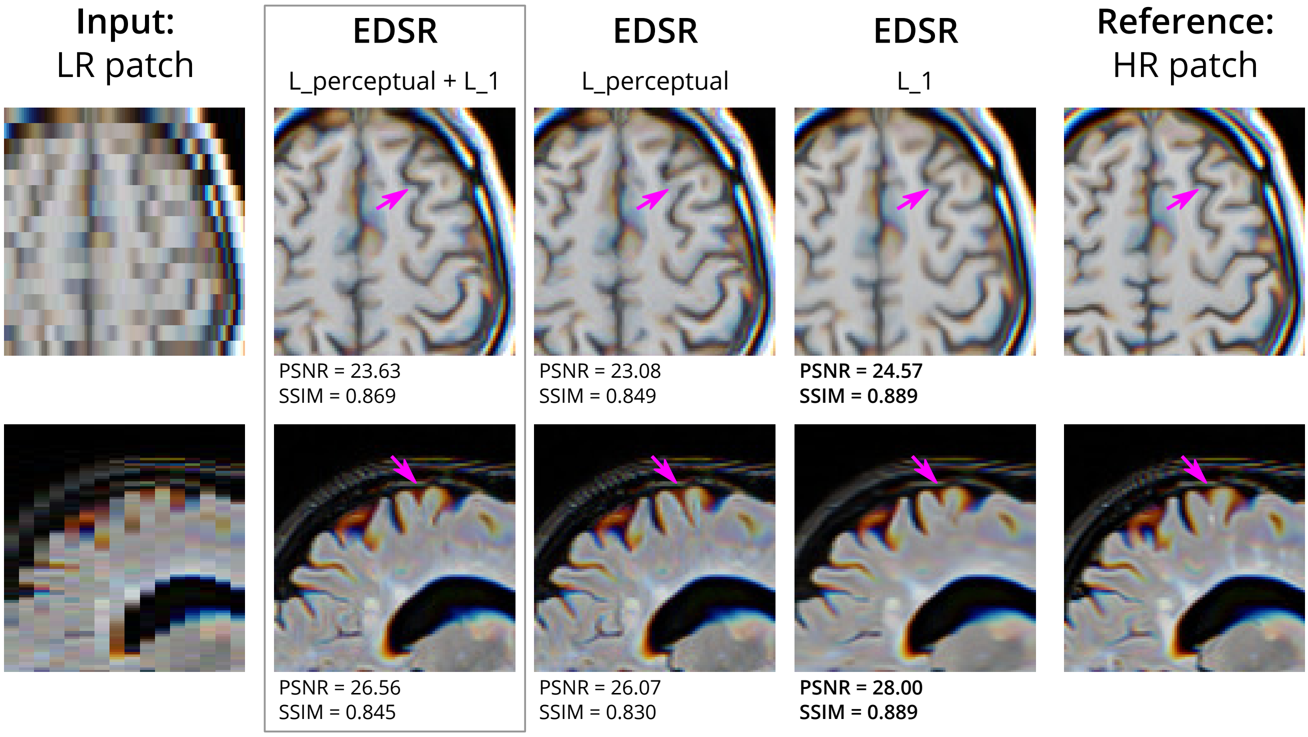

Figure S6. Output examples for EDSR fine-tuned with three different training losses: the content loss used in this work $\mathcal{L}_{\text{perceptual}} + \mathcal{L}_1$ (second column), only $\mathcal{L}_{\text{perceptual}}$ (third column), and only $\mathcal{L}_1$ (fourth column). Images in the third column are sharper than images in the fourth column, but have lower PSNR and SSIM, and exhibit some fabricated structures. Images in the second column are also sharp but closer to the HR reference (fifth column) illustrating that the use of both terms helps to balance perceptual quality and reconstruction accuracy.
